# Causal Relationship Between Metabolic Traits and Risk of NSCLC: A Two-Sample Mendelian Randomization Analysis

**DOI:** 10.7150/jca.109913

**Published:** 2025-09-29

**Authors:** Xin Li, Weifang Cui, Chaojun Duan, Chunfang Zhang

**Affiliations:** 1 Department of Thoracic Surgery, Xiangya Hospital, Central South University, Changsha 410008, Hunan, China.; 2 Hunan Engineering Research Center for Pulmonary Nodules Precise Diagnosis & Treatment, Changsha 410008, Hunan, China.; 3 National Clinical Research Center for Geriatric Disorders, Changsha 410008, Hunan, China.; 4 Xiangya Lung Cancer Center, Xiangya Hospital, Central South University, Changsha 410008, Hunan, China.

**Keywords:** non-small cell lung cancer, metabolic traits, genome-wide association studies, Mendelian Randomization Analysis.

## Abstract

Although the impact of circulating metabolites on the immune microenvironment of lung cancer has been recognized, the causal relationship between these metabolites and non-small cell lung cancer (NSCLC) remains unclear. We investigated whether 233 metabolic traits have a causal effect on NSCLC using data from population-based genome-wide association studies (GWAS). We employed the inverse variance weighted (IVW) method with random effects as the primary analytical tool. After performing two-sample Mendelian randomization (MR) analysis of 233 metabolic traits on NSCLC risk, we selected the significant ones for further sensitivity analyses. Applying a false discovery rate (FDR) correction (P_FDR_ < 0.05), we identified a causal relationship between two metabolic traits and NSCLC: the ratio of Omega-3 fatty acids to total fatty acids (Omega_3_pct) (OR: 1.18, 95% CI: 1.08-1.29, P_FDR_=0.036) and the ratio of 22:6 docosahexaenoic acid to total fatty acids (DHA_pct) (OR: 1.26, 95% CI: 1.11-1.42, P_FDR_=0.036). Sensitivity analyses yielded consistent results. We innovatively utilized GWAS data for multiple metabolic traits to conduct a two-sample Mendelian randomization analysis of the association between 233 metabolic traits and NSCLC. The findings suggest that higher proportions of Omega-3 fatty acids and 22:6 docosahexaenoic acid in total fatty acids may causally increase the risk of NSCLC, providing novel insights into the role of circulating metabolites in lung cancer development.

## Introduction

Lung cancer remains the leading cause of cancer-related mortality worldwide, with approximately 70% of patients diagnosed at either a locally advanced or metastatic stage, resulting in a poor prognosis^1^. NSCLC accounts for nearly 85% of all lung cancer cases^2^. The metabolic processes in rapidly proliferating tumor cells differ significantly from those in healthy cells, resulting in substantial metabolic reprogramming relative to benign tissues^1^, ^3^. This reprogramming involves alterations in key metabolic pathways such as glycolysis, the tricarboxylic acid (TCA) cycle, and fatty acid oxidation^3^. These metabolic changes have important implications in clinical oncology, beyond their fundamental role in cancer biology. Previous studies have primarily relied on case-control or cohort designs, which can identify associations but are limited in inferring causality^4^. Recent findings have established the impact of various circulating blood metabolites, including lactic acid and fatty acids, on tumor growth^5^, ^6^. However, the causal relationship between circulating metabolites and NSCLC remains unclear. Therefore, this study aimed to investigate this association. Investigating the effect of metabolic traits on NSCLC through randomized controlled trials (RCTs), however, is often impractical due to ethical and logistical constraints.

In this framework, Mendelian randomization (MR) serves as a robust epidemiological tool. It utilizes genome-wide association studies (GWAS) and genetic variation as an instrumental variable (IV) to elucidate causal relationships between exposures and outcomes^7^, ^8^. MR is widely regarded as a suitable method for addressing such causal questions. This study implemented a two-sample MR approach, utilizing single nucleotide polymorphisms (SNPs) to evaluate the causal impact of metabolic traits on NSCLC.

## Materials and Methods

### Study design

**Figure 1:**
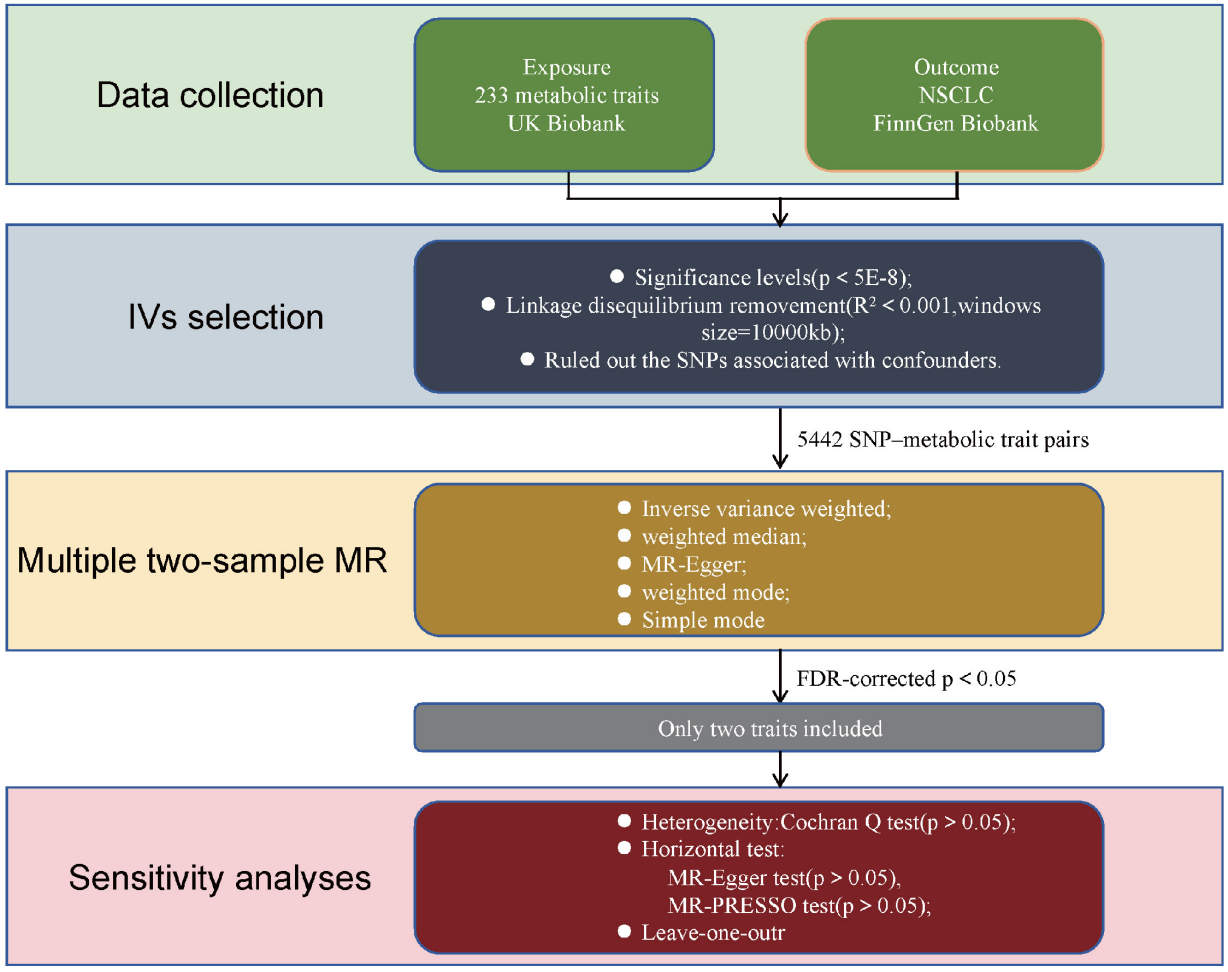
Flowchart illustrating the study design used to investigate the causal effect of 233 metabolic traits on NSCLC. MR Mendelian randomization, SNPs single nucleotide polymorphisms, MR-PRESSO Mendelian Randomization Pleiotropy RESidual Sum and Outlier.

The design of our two-sample Mendelian randomization (MR) study is illustrated in Figure 1. The validity of the MR analyses was evaluated based on three core assumptions: (1) the instrumental variables (IVs) were significantly associated with the metabolic traits; (2) the IVs were not associated with any potential confounders; (3) the IVs affected the outcome exclusively through the exposure of interest^9^.

### Sources of metabolic traits GWAS data

Summary statistics for SNP-metabolic trait associations were obtained from the IEU Open GWAS Project (https://gwas.mrcieu.ac.uk/datasets/?gwas_id__icontains=met-d) and publicly available data from a recent study published in *Nature*^10^. Additional summary statistics for metabolic biomarkers were derived from Nightingale Health (2020) via the UK Biobank initiative (https://www.ukbiobank.ac.uk/learn-more-about-uk-biobank/news/nightingale-health-and-uk-biobank-announces-major-initiative-to-analyse-half-a-million-blood-samples-to-facilitate-global-medical-research). Nightingale Health specializes in health technology, offering a blood analysis platform for large-scale research and personalized health services. Its biomarker profiling technology was used to analyze blood samples from the UK Biobank, measuring metabolic biomarkers that have been associated with the future risk of several common chronic diseases. In total, 233 distinct metabolic traits were quantified from blood samples of approximately 100,000 participants and assessed across ~10 million single nucleotide polymorphisms (SNPs). The conventional genome-wide significance threshold (P < 5E-8) was employed to define associations within the UK Biobank data.

### GAWS data sources for NSCLC

Summary-level data for NSCLC were obtained from the FinnGen Biobank (https://console.cloud.google.com/storage/browser/finngen-public-data-r11/summary_stats/), comprising 5,820 cases and 345,118 cancer-free controls. FinnGen is a large-scale public-private partnership that integrates academic and industry efforts to investigate genetic and environmental factors contributing to common chronic diseases in the Finnish population. It boasts one of the world's largest biobanks, containing genetic data and longitudinal health records from over 500,000 participants. The biobank incorporates comprehensive health information, including electronic medical records, national health registries, and biological samples. Data collection spanned numerous sources, such as hospitals, health centers, and public registries, all linked to genetic information derived from biobank samples. This comprehensive integration facilitates the identification of genetic and environmental contributors to common chronic diseases.

### Selection criteria for IVs for 233 metabolic traits

To investigate the causal relationship between exposure (233 metabolic traits) and outcome (NSCLC), we performed linkage disequilibrium (LD) clustering (r^2^ < 0.001, kb = 10,000) and extracted SNPs significantly associated with each metabolic phenotype (P < 5E-8). We then assessed the strength of the selected instrumental variables (IVs) using the F-statistic. An F-statistic greater than 10 was considered indicative of a strong IV, while metabolic traits with F-statistics below this threshold were excluded^11^. Utilizing these parameters, we identified 5,442 SNP-metabolic trait associations. Next, we extracted the corresponding SNP data from the NSCLC dataset, without using proxy SNPs. A minimum LD threshold of r^2^=0.8 was applied for SNP matching. Exposure and outcome data were synchronized, followed by conducting two-sample Mendelian randomization (MR) analysis.

### Mendelian randomization analysis

We employed three primary Mendelian randomization (MR) methods—inverse variance weighting (IVW), MR-Egger regression, and weighted median—to estimate the causal effects between metabolic traits and NSCLC. The IVW method serves as a meta-analysis approach, assuming that all instrumental variables (IVs) influence the outcome exclusively through the exposure and not via alternative pathways^11^. MR-Egger regression provides consistent causal effect estimates even when all genetic variants exhibit pleiotropy, under the assumption that the association between each genetic variant and the exposure is independent of the pleiotropic effects^12^. The weighted median method yields consistent estimates if at least 50% of the total analysis weight derives from valid instruments^13^. A causal relationship was considered present if any of these methods yielded a statistically significant P value (P < 0.05). For significant findings, sensitivity analyses were performed to assess potential heterogeneity and horizontal pleiotropy. Heterogeneity was evaluated using Cochran's Q test, and horizontal pleiotropy was assessed with the MR-Egger intercept test. An insignificant P value (P > 0.05) in these tests suggests absence of heterogeneity or pleiotropy. Additionally, leave-one-out analyses were conducted by sequentially removing each SNP to evaluate whether any single instrument disproportionately influenced the causal estimate based on IVW.

All analyses were executed in R (version 4.4.0) using the “TwoSampleMR” and “stats” packages. To correct for multiple testing, P_FDR_ were calculated using the Benjamini-Hochberg method, with significance defined as P_FDR_ < 0.05.

## Results

### Multiple two-sample MR analysis of 233 metabolic traits and NSCLC

We conducted a comprehensive two-sample Mendelian randomization (MR) analysis to investigate the associations between 233 metabolic traits—including 213 lipid and lipoprotein parameters, and 20 non-lipid traits such as amino acids and inflammation-related biomarkers—and the risk of NSCLC. Our MR analyses identified four metabolic traits with statistically significant causal associations with NSCLC at the nominal significance threshold (two-sided P < 0.05). These traits included Unsaturation (measuring the estimated degree of unsaturation), Omega_3_pct (the proportion of omega-3 fatty acids relative to total fatty acids), DHA (22:6 docosahexaenoic acid), and DHA_pct (the proportion of DHA relative to total fatty acids). Following the initial MR analyses, we assessed each significant trait for heterogeneity and horizontal pleiotropy (Supplementary files). These findings suggest that fatty acids, particularly unsaturated and omega-3 subtypes, may play a causal role in the development of NSCLC, indicating a positive correlation between unsaturated fatty acid profiles and disease risk.

We subsequently applied a false discovery rate (FDR) correction (P_FDR_ < 0.05) to all Mendelian randomization test results to account for multiple comparisons. After this adjustment, only two metabolic traits remained significantly associated with NSCLC: Omega_3_pct (OR: 1.18, 95% CI: 1.08-1.29, P=2.83E-4, P_FDR_=0.036, Table 1 & Supplementary files) and DHA_pct (OR: 1.26, 95% CI: 1.11-1.42, P=3.19E-4, P_FDR_=0.036, Table 1 & Supplementary files). These results suggest that a higher proportion of Omega-3 fatty acids and docosahexaenoic acid (DHA) relative to total fatty acids may be causally associated with an increased risk of developing NSCLC.

### Further sensitivity analysis of two significant metabolic traits

**Figure 2:**
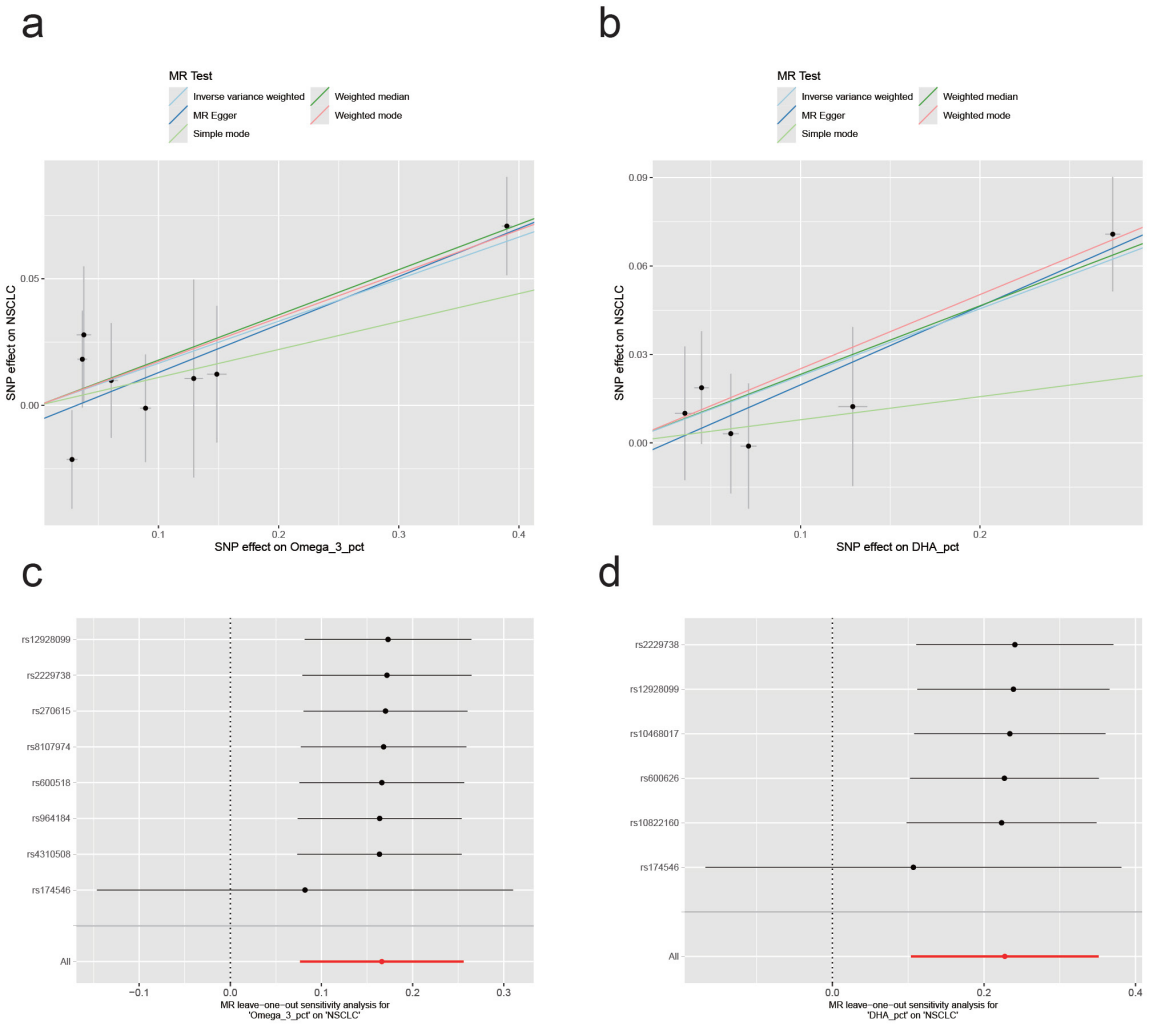
Causal effects of the two significant metabolic traits on NSCLC and results of the leave-one-out sensitivity analysis. (a,b) Scatter plots showing the effect of Omega_3_pct and DHA_pct on NSCLC through genetic variants. (c,d) Leave-one-out plots assessing the robustness of the causal effect estimates for Omega_3_pct and DHA_pct on NSCLC.

Following the identification of two metabolic traits with significant causal associations with NSCLC in our two-sample Mendelian randomization (MR) analysis, we confirmed that two specific traits had significant causal associations. We then conducted comprehensive sensitivity analyses to assess potential heterogeneity and pleiotropy. We employed Cochran's Q-test, MR-Egger's intercept test, and MR-PRESSO for separate assessments of each significant outcome. The results revealed no evidence of heterogeneity or pleiotropy for either Omega_3_pct or DHA_pct: Omega3_pct, Q_Pval=0.75, egger_intercept Pval=0.60, MR-PRESSO Pval=0.73; DHA_pct, Q_Pval=0.83, egger_intercept Pval=0.63, MR-PRESSO Pval=0.71 (Supplementary files). Figure 2 presents the causal effects of the two significant metabolic traits on NSCLC, as revealed by the two-sample Mendelian randomization and leave-one-out analyses. Notably, both associations appear to be predominantly influenced by a single SNP, rs174546. This variant, located within the FADS gene cluster, is a well-established marker of polyunsaturated fatty acid desaturase activity^14^. Further phenotype-wide association analysis (PheWAS) linked rs174546 to Crohn's disease, jaundice, and hypocalcemia. Crohn's disease has been associated with an increased risk of NSCLC (OR=1.53, 95% CI 1.23-1.91, P<0.05)^15^. These findings suggest a notable causal relationship between the two metabolic traits and NSCLC, primarily driven by a single SNP.

In the sensitivity analyses, we observed no evidence of heterogeneity or pleiotropy across all findings. Overall, both Omega-3 and its subtype DHA, as a percentage of total fatty acids, showed a positive causal association with NSCLC. To rule out bidirectional causality, we also conducted reverse MR analyses. A genome-wide P value threshold of 5E-08 was applied for this analysis. The summary statistics for the instrumental SNPs are included in Supplementary files. These analyses revealed no evidence of a causal effect of NSCLC on Omega-3 or DHA levels. Moreover, no heterogeneity or pleiotropy was detected in the reverse MR (Supplementary files).

## Discussion

In this study, we performed extensive two-sample MR analyses of the causal impacts of metabolic traits on NSCLC. Our findings suggest significant causal associations between polyunsaturated fatty acids and NSCLC, specifically unsaturation levels, docosahexaenoic acid (DHA, 22:6), Omega_3_pct (the ratio of omega-3 fatty acids to total fatty acids), and DHA_pct (the ratio of 22:6 docosahexaenoic acid to total fatty acids). These unsaturated fatty acids, sourced from dietary consumption, serve as crucial precursors for eicosanoid hormones and modulate various processes linked to cancer and other diseases such as inflammation, thrombosis, and insulin resistance^16^, ^17^.

After correcting for multiple testing using the FDR, only two phenotypes remained statistically significant: Omega3_pct (OR: 1.18, 95%CI: 1.08-1.29, P=2.83e-4, P_FDR_=0.036) and DHA_pct (OR: 1.26, 95%CI: 1.11-1.42, P=3.19e-4, P_FDR_=0.036). Previous observational studies and meta-analyses have explored potential protective associations between omega-3 PUFAs and cancer risk in general^18^-^21^. However, evidence regarding site-specific cancers such as lung cancer remains limited and inconsistent. A 2022 MR study investigating the causal effects of omega-3 PUFAs on lung cancer did not find significant associations, likely due to its narrower metabolic scope and the exclusion of related lipid traits^22^. In contrast, our comprehensive approach encompassing 233 metabolic phenotypes suggests that a higher omega-3 to total fatty acid ratio may increase lung cancer risk. Notably, other data suggest that higher intake of unsaturated fatty acids may increase cancer risk^23^, ^24^. A study demonstrated a causal relationship between increased biosynthesis of PUFAs and the risk of colorectal and esophageal squamous cell carcinomas, suggesting a carcinogenic role for PUFAs^14^. Overall, our study highlights a robust, FDR-significant causal link between increased omega-3 fatty acid ratios and NSCLC risk, with this relationship appearing to be largely driven by the SNP rs174546, a known regulator of PUFA metabolism. These findings challenge the widely held assumption that omega-3 fatty acids are uniformly protective and suggest a more nuanced role in lung cancer development.

Omega-3 fatty acids are essential for human health and play important roles in a variety of diseases, making them critical components in the body^25^. The main Omega-3 fatty acids include the essential fatty acid α-linolenic acid (ALA), as well as eicosapentaenoic acid (EPA) and docosahexaenoic acid (DHA), both of which are synthesized endogenously from ALA^26^, ^27^. Omega-3 fatty acids are incorporated into multiple tissues, with DHA serving as a major structural component of cell membranes, particularly in neural and retinal tissues^28^. Our study suggests that the ratios of total Omega-3 fatty acids and DHA to total fatty acids may represent causal risk factors for NSCLC. Sensitivity analyses showed no evidence of heterogeneity or pleiotropy. Importantly, leave-one-out analyses indicated that the positive causal associations between total Omega-3 and DHA fatty acids and NSCLC risk were largely driven by the genetic variant rs174546 located in the FADS gene locus. The FADS gene encodes the delta-5 (D5D) and delta-6 (D6D) desaturase enzymes, which catalyze the rate-limiting desaturation steps in the biosynthesis of Omega-3 polyunsaturated fatty acids^14^. Therefore, the ratios of Omega-3 and DHA fatty acids to total fatty acids may influence NSCLC development through the activity of unsaturated fatty acid desaturases regulated by the FADS gene.

One potential molecular mechanism involves increased synthesis of arachidonic acid mediated by delta-5 desaturase (D5D), which is encoded by the FADS gene. Arachidonic acid is a key substrate for cyclooxygenase enzymes (COX-1 and COX-2), leading to the production of pro-inflammatory and pro-tumorigenic eicosanoids, notably prostaglandin E2 (PGE2)^29^-^31^. These metabolites possess potent pro-inflammatory properties, linking them to a heightened risk of inflammatory disorders such as Crohn's disease, as well as to cancer development. Furthermore, the upregulated synthesis and metabolism of arachidonic acid may promote tumorigenesis by enhancing cell proliferation and fostering a pro-inflammatory microenvironment that facilitates oncogenic mutations^29^-^31^. These findings are consistent with epidemiological evidence showing that non-steroidal anti-inflammatory drugs (NSAIDs), such as aspirin, reduce cancer risk by inhibiting cyclooxygenase-mediated arachidonic acid metabolism, thereby lowering the incidence of colorectal cancer and potentially other malignancies.

To our knowledge, this study is the first to systematically investigate the direct causal associations between a broad spectrum of metabolic traits and NSCLC risk using Mendelian randomization. Our analyses identified that a higher proportion of omega-3 polyunsaturated fatty acids (PUFAs) relative to total fatty acids may serve as a potential risk factor for NSCLC development among the 233 metabolic traits examined. These findings suggest that modulating the biosynthesis or dietary intake of omega-3 PUFAs could represent a potential strategy for NSCLC prevention^32^-^34^. For example, preventive approaches might include cautious consideration of omega-3 rich food consumption alongside the use of non-steroidal anti-inflammatory drugs (NSAIDs), such as aspirin, which inhibit cyclooxygenase (COX)-mediated arachidonic acid metabolism^34^, ^35^. Current dietary guidelines generally recommend replacing saturated fats with unsaturated fatty acids to reduce cardiovascular disease risk. However, our results imply that individuals at elevated risk for NSCLC may need to approach these recommendations with caution.

This study focused on a comprehensive set of 233 metabolic traits, primarily encompassing common metabolic phenotypes. We confirmed a positive causal relationship between the ratio of omega-3 unsaturated fatty acids to total fatty acids and NSCLC risk. However, certain metabolic traits, such as serum acetylcholine levels, were not included due to limitations in available GWAS data. Sensitivity analyses revealed no significant heterogeneity or pleiotropy, although leave-one-out analyses indicated that the observed causal effects were largely driven by a single genetic variant, rs174546, located within the FADS gene region. This variant likely affects desaturase enzyme activity, which may impact the robustness of our findings. Overall, our study benefits from a large sample size and stringent false discovery rate (FDR) correction for multiple comparisons, enhancing the reliability of the results. Nevertheless, the Finnish population, characterized by a genetic bottleneck and relative distinctiveness from other European populations, warrants cautious generalization of our findings^6^. Furthermore, we were unable to validate our primary findings using surgically resected human NSCLC tissue samples. Future studies should aim to employ internal or external validation methods, commonly applied in experimental settings, to corroborate these results.

## Conclusion

The ratio of Omega-unsaturated fatty acids to total fatty acids is positively and causally associated with the risk of NSCLC. Therefore, targeting the biosynthetic pathway of Omega-3 unsaturated fatty acids may represent a potential strategy for NSCLC prevention.

## Supplementary Material

Supplementary data.

## Figures and Tables

**Table 1:** Details of two significant traits after multiple two-sample MR test with false discovery rate P-value (P_FDR_<0.05).

	Outcome	Method	SNPs	Beta	Se	OR (95%CI)	P value	P_FDR_	P_Heterogeneity_	P_Pleiotropy_
Omega_3_pct	NSCLC	MR Egger	8	0.19	0.06	1.21 (1.07-1.37)	2.33e-02		0.75	0.60
		Weighted median		0.18	0.05	1.20 (1.09-1.32)	2.32e-04			
		IVW		0.17	0.05	1.18 (1.08-1.29)	2.83e-04	0.036	0.81	
		Simple mode		0.11	0.12	1.12 (0.88-1.41)	3.86e-01			
		Weighted mode		0.17	0.05	1.19 (1.08-1.31)	8.50e-03			
		MR PRESSO					0.73			
DHA_pct	NSCLC	MR Egger	6	0.27	0.10	1.31 (1.08-1.58)	5.38e-02		0.83	0.63
		Weighted median		0.23	0.07	1.26 (1.10-1.44)	7.19e-04			
		IVW		0.23	0.06	1.26 (1.11-1.42)	3.19e-04	0.036	0.88	
		Simple mode		0.08	0.14	1.08 (0,81-1.44)	6.11e-01			
		Weighted mode		0.25	0.07	1.29 (1.11-1.48)	1.81e-02			
		MR PRESSO					0.71			

Se standard error; FDR false discovery rate; IVW inverse variance weighted.
