# Supplementary material for: Causal Relationship Between Metabolic Traits and Risk of NSCLC: A Two-Sample Mendelian Randomization Analysis
Source: J Cancer. 2025 Sep 29;16(14):4139–46. doi: 10.7150/jca.109913 (PMC12595243; doi:10.7150/jca.109913)
Supplement: Supplementary file 1 — Supplementary data. [file jcav16p4139s1.pdf]

## MR results of two metabolic traits with NSCLC

### Omega_3_pct SNP

| SNP | effect_al | other_all | effect_al | other_all | beta.expc | beta.outc | eaf.expos | eaf.outcc |
|---|---|---|---|---|---|---|---|---|
| rs1292809 | A | C | A | C | 0.089249 | -0.0011 | 0.2883 | 0.281411 |
| rs174546 | T | C | T | C | -0.38996 | -0.07082 | 0.3469 | 0.4141 |
| rs2229738 | T | C | T | C | -0.14857 | -0.01232 | 0.0865 | 0.155919 |
| rs270615 | C | G | C | G | 0.02805 | -0.02136 | 0.6769 | 0.584042 |
| rs4310508 | G | A | G | A | 0.036655 | 0.018226 | 0.4871 | 0.472888 |
| rs600518 | T | A | T | A | -0.06066 | -0.00986 | 0.171 | 0.230482 |
| rs8107974 | T | A | T | A | -0.12936 | -0.01062 | 0.0686 | 0.0636 |
| rs964184 | C | G | C | G | -0.03792 | -0.02785 | 0.838 | 0.855372 |

### Omega_3_pct results

| exposure | outcome | method | nsnp | b | se | pval | lo_ci | up_ci |
|---|---|---|---|---|---|---|---|---|
| Omega_3_p | NSCLC | MR Egger | 8 | 0.189744 | 0.062752 | 0.023285 | 0.066751 | 0.312738 |
| Omega_3_p | NSCLC | Weighted | 8 | 0.178701 | 0.04855 | 0.000233 | 0.083544 | 0.273859 |
| Omega_3_p | NSCLC | Inverse v | 8 | 0.166186 | 0.045776 | 0.000283 | 0.076465 | 0.255907 |
| Omega_3_p | NSCLC | Simple mc | 8 | 0.110355 | 0.119386 | 0.386059 | -0.12364 | 0.344353 |
| Omega_3_p | NSCLC | Weighted | 8 | 0.173242 | 0.047847 | 0.008501 | 0.079462 | 0.267021 |

### Omega_3_pct heterogeneity

| exposure | outcome | method | Q | Q_df | Q_pval |
|---|---|---|---|---|---|
| Omega_3_p | NSCLC | MR Egger | 3.470295 | 6 | 0.747918 |
| Omega_3_p | NSCLC | Inverse v | 3.771542 | 7 | 0.805683 |

### Omega_3_pct pleiotropy

| exposure | outcome | egger_int | se | pval |
|---|---|---|---|---|
| Omega_3_p | NSCLC | -0.00603 | 0.010983 | 0.602911 |

### Omega_3_pct MR-PRESSO

| | RSSobs | Pvalue |
|---|---|---|
| 1 | 7.785915 | 0.731 |

### DHA_pct SNP

| SNP | effect_al | other_all | effect_al | other_all | beta.expc | beta.outc | eaf.expos | eaf.outcc |
|---|---|---|---|---|---|---|---|---|
| rs1046801 | T | C | T | C | 0.061109 | 0.003126 | 0.2833 | 0.331083 |
| rs1082216 | G | T | G | T | 0.044815 | 0.018716 | 0.4861 | 0.472735 |
| rs1292809 | A | C | A | C | 0.070977 | -0.0011 | 0.2883 | 0.281411 |
| rs174546 | T | C | T | C | -0.27403 | -0.07082 | 0.3469 | 0.4141 |
| rs2229738 | T | C | T | C | -0.12909 | -0.01232 | 0.0865 | 0.155919 |
| rs600626 | G | A | G | A | -0.03552 | -0.01002 | 0.171 | 0.230569 |

### DHA_pct MRresults

| exposure | outcome | method | nsnp | b | se | pval | lo_ci | up_ci |
|---|---|---|---|---|---|---|---|---|
| DHA_pct | NSCLC | MR Egger | 6 | 0.266491 | 0.098484 | 0.053762 | 0.073464 | 0.459519 |
| DHA_pct | NSCLC | Weighted | 6 | 0.232508 | 0.068746 | 0.000719 | 0.097765 | 0.36725 |
| DHA_pct | NSCLC | Inverse v | 6 | 0.227516 | 0.063214 | 0.000319 | 0.103617 | 0.351414 |
| DHA_pct | NSCLC | Simple mc | 6 | 0.078388 | 0.144634 | 0.611104 | -0.20509 | 0.36187 |
| DHA_pct | NSCLC | Weighted | 6 | 0.251538 | 0.0728 | 0.018136 | 0.108849 | 0.394227 |

**DHA_pct heterogeneity**

| exposure | outcome | method | Q | Q_df | Q_pval |
|---|---|---|---|---|---|
| DHA_pct | NSCLC | MR Egger | 1.469323 | 4 | 0.832061 |
| DHA_pct | NSCLC | Inverse v | 1.735692 | 5 | 0.884373 |

**DHA_pct pleiotropy**

| exposure | outcome | egger_int | se | pval |
|---|---|---|---|---|
| DHA_pct | NSCLC | -0.00696 | 0.013489 | 0.632997 |

**DHA_pct MR-PRESSO**

| | RSSobs | Pvalue |
|---|---|---|
| 1 | 6.297764 | 0.713 |

**Reverse MR results of two metabolic traits with NSCLC**

**NSCLC and Omega3_pct MR result**

| SNP | effect_al | other_all | effect_al | other_all | beta.expc | beta.outc | eaf.expos | eaf.outcc |
|---|---|---|---|---|---|---|---|---|
| rs1291438 | T | C | T | C | 0.250651 | -0.00315 | 0.356526 | 0.382525 |
| rs3590050 | A | C | A | C | 0.119163 | 0.002285 | 0.25565 | 0.2924 |
| rs37003 | C | A | C | A | -0.13644 | 0.00309 | 0.439149 | 0.440911 |
| rs6011779 | T | C | T | C | -0.12542 | 0.003899 | 0.755965 | 0.808887 |
| rs8066706 | C | T | C | T | 0.419587 | -0.00219 | 0.017157 | 0.020112 |

**NSCLC and DHA_pct MR result**

| SNP | effect_al | other_all | effect_al | other_all | beta.expc | beta.outc | eaf.expos | eaf.outcc |
|---|---|---|---|---|---|---|---|---|
| rs1291438 | T | C | T | C | 0.250651 | -0.00685 | 0.356526 | 0.382525 |
| rs3590050 | A | C | A | C | 0.119163 | 0.005629 | 0.25565 | 0.2924 |
| rs37003 | C | A | C | A | -0.13644 | -0.00132 | 0.439149 | 0.440911 |
| rs6011779 | T | C | T | C | -0.12542 | 0.003532 | 0.755965 | 0.808887 |
| rs8066706 | C | T | C | T | 0.419587 | 0.008486 | 0.017157 | 0.020112 |

**NSCLC-Omega_3_pct heterogeneity**

| exposure | outcome | method | Q | Q_df | Q_pval |
|---|---|---|---|---|---|
| NSCLC | Omega3_pc | MR Egger | 4.008363 | 3 | 0.260563 |
| NSCLC | Omega3_pc | Inverse v | 4.827535 | 4 | 0.305456 |

**NSCLC-DHA_pct heterogeneity**

| exposure | outcome | method | Q | Q_df | Q_pval |
|---|---|---|---|---|---|
| NSCLC | DHA_pct | MR Egger | 1.061459 | 3 | 0.786385 |
| NSCLC | DHA_pct | Inverse v | 1.061498 | 4 | 0.900332 |

**NSCLC-Omega3_pct pleiotropy**

| exposure | outcome | egger_int | se | pval |
|---|---|---|---|---|
| NSCLC | Omega3_pc | 0.005312 | 0.006784 | 0.490748 |

**NSCLC-DHA_pct pleiotropy**

| exposure | outcome | egger_int | se | pval |
|---|---|---|---|---|
| NSCLC | DHA_pct | 3.71E-05 | 0.005924 | 0.995394 |

| remove | pval.outc | se.outcom | outcome | exposure | se.exposu | pval.exposu | mr_keep |
|---|---|---|---|---|---|---|---|
| FALSE | 0.958596 | 0.021254 | NSCLC | Omega_3_p | 0.004458 | 8.20E-90 | TRUE |
| FALSE | 0.000265 | 0.019415 | NSCLC | Omega_3_p | 0.004242 | 1.00E-200 | TRUE |
| FALSE | 0.647979 | 0.026974 | NSCLC | Omega_3_p | 0.008099 | 1.10E-76 | TRUE |
| FALSE | 0.272402 | 0.019463 | NSCLC | Omega_3_p | 0.004473 | 4.30E-11 | TRUE |
| FALSE | 0.341165 | 0.019148 | NSCLC | Omega_3_p | 0.004058 | 7.10E-21 | TRUE |
| FALSE | 0.663419 | 0.022665 | NSCLC | Omega_3_p | 0.005555 | 7.10E-29 | TRUE |
| FALSE | 0.785684 | 0.039058 | NSCLC | Omega_3_p | 0.007693 | 8.60E-67 | TRUE |
| FALSE | 0.302641 | 0.027023 | NSCLC | Omega_3_p | 0.005947 | 1.20E-10 | TRUE |

| or | or_lci95 | or_uci95 |
|---|---|---|
| 1.208941 | 1.069029 | 1.367163 |
| 1.195664 | 1.087133 | 1.31503 |
| 1.180793 | 1.079464 | 1.291633 |
| 1.116674 | 0.883696 | 1.411076 |
| 1.189154 | 1.082705 | 1.306068 |

| remove | pval.outc | se.outcom | outcome | exposure | se.exposu | pval.exposu | mr_keep |
|---|---|---|---|---|---|---|---|
| FALSE | 0.877674 | 0.020312 | NSCLC | DHA_pct | 0.004409 | 6.40E-45 | TRUE |
| FALSE | 0.328325 | 0.019146 | NSCLC | DHA_pct | 0.00402 | 5.50E-30 | TRUE |
| FALSE | 0.958596 | 0.021254 | NSCLC | DHA_pct | 0.004417 | 2.60E-59 | TRUE |
| FALSE | 0.000265 | 0.019415 | NSCLC | DHA_pct | 0.004214 | 1.00E-200 | TRUE |
| FALSE | 0.647979 | 0.026974 | NSCLC | DHA_pct | 0.008046 | 9.70E-58 | TRUE |
| FALSE | 0.658312 | 0.022666 | NSCLC | DHA_pct | 0.005518 | 6.20E-11 | TRUE |

| or | or_lci95 | or_uci95 |
|---|---|---|
| 1.305376 | 1.076229 | 1.583313 |
| 1.26176 | 1.102704 | 1.443759 |
| 1.255477 | 1.109176 | 1.421076 |
| 1.081543 | 0.814571 | 1.436012 |
| 1.286002 | 1.114994 | 1.483237 |

| se.outcom | pval.outc | pval.expc | se.exposu | mr_keep |
|---|---|---|---|---|
| 0.004171 | 0.450001 | 3.24E-38 | 0.019392 | TRUE |
| 0.004488 | 0.59 | 3.06E-08 | 0.021518 | TRUE |
| 0.004119 | 0.56 | 1.91E-12 | 0.019377 | TRUE |
| 0.005174 | 0.28 | 7.27E-09 | 0.021681 | TRUE |
| 0.014591 | 0.95 | 4.27E-10 | 0.067199 | TRUE |

| se.outcom | pval.outc | pval.expc | se.exposu | mr_keep |
|---|---|---|---|---|
| 0.004132 | 0.11 | 3.24E-38 | 0.019392 | TRUE |
| 0.004446 | 0.21 | 3.06E-08 | 0.021518 | TRUE |
| 0.00408 | 0.649999 | 1.91E-12 | 0.019377 | TRUE |
| 0.005125 | 0.42 | 7.27E-09 | 0.021681 | TRUE |
| 0.014453 | 0.42 | 4.27E-10 | 0.067199 | TRUE |
